# Does CD4+CD25+foxp3+ cell (Treg) and IL-10 profile determine susceptibility to immune reconstitution inflammatory syndrome (IRIS) in HIV disease?

**DOI:** 10.1186/1476-9255-5-2

**Published:** 2008-02-18

**Authors:** Esaki Muthu Shankar, Ramachandran Vignesh, Vijayakumar Velu, Kailapuri G Murugavel, Ramalingam Sekar, Pachamuthu Balakrishnan, Charmaine AC Lloyd, Shanmugam Saravanan, Suniti Solomon, Nagalingeswaran Kumarasamy

**Affiliations:** 1 YRG Centre for AIDS Research and Education, VHS Hospital Campus, Rajiv Gandhi Salai-Information Technology Corridor, Taramani, Chennai 600 113, India

## Abstract

HIV-specific T-lymphocyte responses that underlie IRIS are incomplete and largely remain hypothetical. Of the several mechanisms presented by the host to control host immunological damage, Treg cells are believed to play a critical role. Using the available experimental evidence, it is proposed that enormous synthesis of conventional FoxP3_- _Th cells (responsive) often renders subjects inherently vulnerable to IRIS, whereas that of natural FoxP3^+ ^Treg cell synthesis predominate among subjects that may not progress to IRIS. We also propose that IRIS non-developers generate precursor T-cells with a high avidity to generate CD4+CD25+FoxP3+ Tregs whereas IRIS developers generate T-cells of intermediate avidity yielding Th0 cells and effector T-cells to mediate the generation of proinflammatory cytokines in response to cell-signaling factors (IL-2, IL-6 etc.). Researchers have shown that IL-10 Tregs (along with TGF-β, a known anti-inflammatory cytokine) limit immune responses against microbial antigens in addition to effectively controlling HIV replication, the prime objective of HAART. Although certain technical limitations are described herein, we advocate measures to test the role of Tregs in IRIS.

## The hide and seek game in immune reconstitution inflammatory syndrome (IRIS): The factor?

The immune reconstitution inflammatory syndrome (IRIS) in HIV-infected patients initiating highly active antiretroviral therapy (HAART) leading to 'paradoxical clinical worsening' [1] results from restored immunity to specific infectious or non-infectious antigens [2-10]. Possible mechanisms include a partial recovery of the host immune system or exuberant immunological responses to antigenic stimuli. The overall frequency of IRIS is undefined, but is believed to be dependent on underlying opportunistic infectious (mycobacteria, varicella zoster, herpesviruses, and cytomegalovirus) and non-infectious (autoimmune) burdens [2-10]. Of the subjects that are initiated on HAART, only a proportion progress to develop IRIS and the remaining never develop IRIS despite an exuberant immune restoration from poor baseline CD4+ T-cell levels. Recently, we proposed that subjects that develop IRIS generate a high burden of proinflammatory cytokines in response to enormous levels of systemic bacterial LPS as compared with less LPS in IRIS non-developers [11]. Although several other mechanisms have been proposed, HIV-specific T-cell responses underlying IRIS are incomplete [12]. To investigate the inflammatory intermediaries of IRIS it will be crucial to explain the intrinsic dynamics of immune cells after initiating HAART [13]. Furthermore, factors that confers resistance to development of IRIS among IRIS non-developers needs to be described.

## Possible role of memory T-cells in IRIS

The majority of individuals with HIV infection in resource-constrained settings attend HIV testing centres only after progression to terminal stage of HIV disease (i.e. when their CD4+ T-cell counts are very low) [1,14]. Therefore, these subjects with the most severe immunosuppression (CD4+ T-cell nadirs, 100 cells/μL), initiating antiretroviral regimens may be at the highest risk for the development of IRIS [15]. Conceptually, any individual harboring microbial antigens should mount an over-exuberant immune response against any pre-existing antigen (e.g. mycobacterial, cryptococcal, strongyloidal, etc.), which at times may be detrimental to the host owing to subsequent inflammatory reactions that follow the restoration.

Reconstitution of memory cell responses is the initial hallmark of restoration of pathogen specific immune responses after initiation of HAART. Recent evidence suggests that the immune restoration following HAART is characterized by enormous memory CD4+ T-cell types (CD4+CD45RO+) likely due to peripheral lymphoid redistribution [16,17]. A characteristic 2-phase increase in CD4 T-cells occurs after initiating HAART; a rapid initial increase in memory T cells in the first few months, followed by a steady rise in naive T-cells that continue for years with sustained therapy. Furthermore, recovery of lost responses occurring during the early phase could also be attributed to cellular redistribution rather than a *de novo *specific CD4+ T-cell proliferation since naive activated CD4+ T-cells (CD4+CD45RA+CD62L+) do not recover until after several months of therapy. The feature and strength of this response may account the early-onset of IRIS [3]. Moreover, an increase in the concentrations of IFN-γ and IL-2 are reportedly responsible for this phenomenon. There are a few studies to support the proliferation of peripheral blood mononuclear cells, antigen specific CD4+ T-cells and IFN-γ response to *Mycobacterium tuberculosis *antigens after initiation of HAART in HIV-infected patients [18,19]. The activated CD4+ T-cells are subsequently primed to recognize previous antigenic stimuli, and might account for the subsequent manifestations of IRIS. This, possibly could be due to the fact that generation of post-HAART immune components always arise from residual immune cells that had undergone extensive immune activation rather than the newly synthesized cells. Researchers have also shown that improvement in antigen-specific T-cell restoration could be attributed to an increase in memory CD4+ T-cell number but not with increase in CD4+ T-cell number or decrease in plasma viral load (PVL) [20]. Further, the study has shown that improvement in delayed type hypersensitivity (DTH) response was significantly associated only with a decline in PVL [20].

## IRIS developers vs IRIS non-developers: Are Tregs the determinants of inflammation after initiating HAART?

Of the several mechanisms put forth by the host to regulate immunological damage caused by over-exuberant immune responses, Treg cells are believed to play a critical role in regulating inflammatory responses by mediating key components that facilitate immune suppression [21-25]. Of the various Treg populations described, the CD4^+^CD25^+ ^and CD4^+^CD45RB^low ^cells function via the action of IL-10 [26], cytotoxic T-cell-associated protein 4 (CTLA4) [27,28], and/or TGF-β [26-33], while other Tregs secrete IL-10 [31-40] and TGF-β [41]. Tregs and naïve helper T cells (Th0) are proposed to develop within a normal thymus through positive and negative selection processes. On the one hand, it is proposed that enormous generation of conventional FoxP3_- _Th cells (CD5^low^, CD11a^low^, CD25^low^, CD38^low^, CD44^low^, CD45RB^high^, CD54^low^, CD103ε ^low^, GITR^low^), that reportedly are panergic to T-cell stimuli and non-suppressive, predominate among subjects that are inherently vulnerable to IRIS. On the other hand, the high turnover of natural FoxP3^+ ^Treg cells (CD5^high^, CD11a^high^, CD25^high^, CD38^high^, CD44^high^, CD45RB^low^, CD54^high^, CD103^high^, GITR^high^), that are conventionally non-responsive to T-cell stimuli and suppressive (normally beneficial, as it may help limit the severity of tissue destruction associated with an inflammatory condition due to infection), predominate among subjects that may not progress to IRIS. Therefore, precursor T-cells of relatively high avidity trigger Treg development via the activation of Foxp3 (forkhead/winged-helix family transcriptional repressor), whereas T-cell receptors of intermediate avidity yield conventional Th0 cells. We propose that IRIS non-developers generate precursor T-cells with a high avidity to generate CD4+CD25+FoxP3+ Tregs whereas IRIS developers generate T-cells of intermediate avidity yielding Th0 cells and effector T-cells to mediate the generation of proinflammatory cytokines in response to cell-signaling factors (IL-2, IL-6 etc.). We propose that IRIS non-developers possess Foxp3^+ ^in the naturally occurring Tregs that inhibit CD4^+ ^T-cell proliferation through IL-10-independent, cell contact-dependent mechanisms. This mechanism has already been demonstrated in certain other inflammatory conditions. Besides this, plasmacytoid dendritic cells (pDC) expressing ICOS ligand (ICOS-L) appears to be responsible for the generation of IL-10 Tregs. Tregs are known to suppress cellular proliferation and cytokine production by CD4^+ ^and CD8^+ ^T-cells in response to microbial antigens. Researchers have shown that IL-10 levels are raised after HAART among IRIS developers [13,42]. Furthermore, IL-10 Tregs also limit immune responses against microbial antigens (*M. tuberculosis*) in addition to controlling HIV replication [42,43]. Tregs also suppress HIV-induced proliferation of perforin-expressing CD8^+ ^T-cells, which otherwise, could mediate inflammation [42,44,45]. Both Th1 and Th2 responses can be controlled by IL-10 Tregs and by naturally occurring Tregs [46]. It is to be remembered that Th1 cells producing IFN-γ are critical for obliterating intracellular pathogens, notably *M. tuberculosis *and *Listeria monocytogenes*, but are also implicated in inflammatory pathologies [47,48]. Th2 cells, on the other hand, which produce IL-4, IL-5, and IL-13, are important for the regulation of immune responses to helminths but also cause allergic pathologies [47,49]. IL-4 and IL-13 are implicated in inhibiting the generation of proinflammatory cytokines by macrophages [49]. In addition to being regulated by naturally occurring Tregs and by IL-10 Tregs, Th1 and Th2 cells also reciprocally regulate the development and function of each other. Because IL-10 is also produced by B lymphocytes, macrophages, DCs and T cells other than Tregs, it is clear that multiple cell types could contribute to the regulation of host immune responses via their production of IL-10. IL-10 is reported to be essential in limiting immune responses to inflammation [42,50]. Research on experimental animals has shown that the deletion of IL-10 gene could facilitate susceptibility to inflammatory bowel disease and colitis [49,50]. Thus regulation of the immune response may occur at various levels, each utilizing different mechanisms that, depending on the degree of inflammation and the host response to infection, are called upon at different stages during immune responses. The regulatory capacity of antigen-driven IL-10 Tregs and of naturally occurring Tregs and the induction of immunosuppressive cytokines (IL-10 and TGF-β) provide a new level of immune regulation to inhibit both Th1- and Th2-type immune responses [50]. Treg fitness could be another possible approach that appears to be of interest with regard to Treg levels in chronic HIV disease. Although, we propose to test the immunosuppressive role of Tregs in IRIS, we do foresee certain limitations for testing the hypotheses. While CD4/CD25/FoxP3 are well-cited markers for Tregs, there appears to be a consensus in the flow cytometry community on which markers actually define the Treg population (activated and non-activated) in the presence of HIV infection. Furthermore, Tregs are often reported to be few in numbers in flow cytometric analyses and therefore in the setting of HIV infection, it is questionable as to whether the frequency of these cells is sufficient for testing the proposed hypothesis. Future attempts using sophisticated tools and technologies might render this possible. We also warrant future research required to reveal the mechanisms that underlie the possible role of APCs, effector molecules, such as IL-10 and TGF-β, secreted by Tregs in determining the susceptibility of individuals to IRIS.

## Conclusion

We propose to test whether IRIS non-developers generate precursor T-cells with a high avidity to generate CD4+CD25+FoxP3+ Tregs and whether IRIS developers generate T-cells of intermediate avidity yielding Th0 cells and effector T-cells. Although researchers have shown that IL-10 Tregs control immune responses against foreign antigens in addition to effectively controlling HIV replication, the prime objective of HAART, this needs to be intensively investigated. Attempts have never been thrown to reveal the role of tregs in IRIS and FoxP3 gene till date. The hypothesis can be either tested in a disease-specific approach (i.e. examining the role of Tregs in TB-IRIS subjects) or by examining all forms of IRIS (both infectious and non-infectious). In addition, the role of polymorphisms in TNF-α and IL-6 promotors can also be examined as these too could play a significant role in mediating IRIS.

## Competing interests

The author(s) declare that they have no competing interests.

## Authors' contributions

EMS, RV, VV and NK conceived and proposed the hypothesis. RV, KGM, RS, PB, CACL, SSo, and NK provided additional inputs to further develop the scientific concept; EMS, RV, SSa, RS and PB drafted the manuscript; SSo and NK shared their clinical expertise and critically revised the manuscript. All authors read and approved the final manuscript. EMS, RV and NK are the guarantors of the paper.
